# Supplementary figures and images for: Copper Death Inducer, FDX1, as a Prognostic Biomarker Reshaping Tumor Immunity in Clear Cell Renal Cell Carcinoma
Source: Cells. 2023 Jan 17;12(3):349. doi: 10.3390/cells12030349 (PMC9913648; doi:10.3390/cells12030349)

Figure S1

A

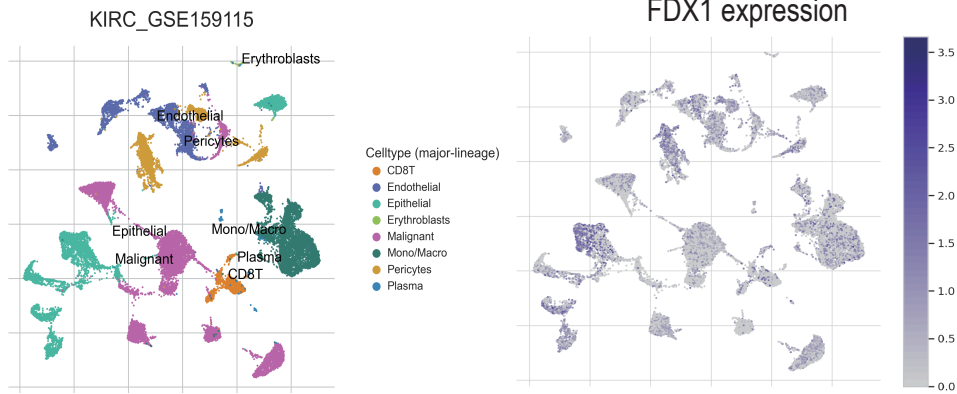

B

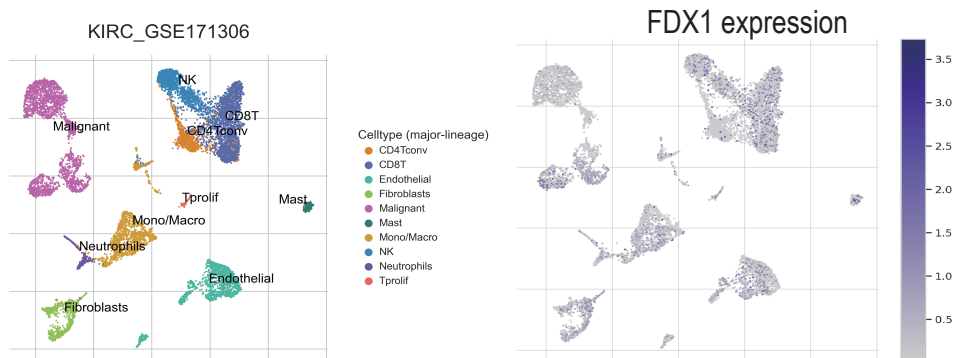

Supplement: Supplementary file 1 [file cells-12-00349-s001.zip › cells-2109105-supplementary/FigureS1.pdf]
